# Mitochondrial NADK, a Novel Functional Mediator of Tau Toxicity in Alzheimer's Disease

**DOI:** 10.1002/alz70856_110903

**Published:** 2026-01-09

**Authors:** Andrés Norambuena

**Affiliations:** ^1^ University of Virginia, Charlottesville, VA, USA

## Abstract

Tau was first described in a 1975 PNAS paper from Marc Kirschner's lab as a brain protein that stimulates the polymerization of tubulin into microtubules (https://doi.org/10.1073/pnas.72.5.1858). Unbeknown at that time was the fact that tau is the building block of neurofibrillary tangles, and a central player in the pathogenesis of Alzheimer's disease (AD) and the numerous non‐Alzheimer's tauopathies. Tau's discovery thus serves as a textbook example of how advances in basic science lie at the foundation of clinical medicine. To commemorate the 50th anniversary of the first publication about tau, this Featured Research Session will highlight the work of 6 speakers, spanning basic to clinical scientists, and those who bridge the gap between these opposite, but complementary ends of the AD research community. The talks will cover major advances in tau research and how they have influenced the AD field. They will include a discussion on the complex structural and molecular heterogeneity of tau proteins, mechanisms mediating its toxicity and spread, its clinical utility as both an imaging and fluid biomarker, and innovative therapeutic strategies to mitigate its toxicity. Talks will be delivered by Michel Goedert, Keith Johnson, Bess Frost, Henrik Zetterberg. and the co‐organizers, Miranda Orr and George Bloom. Each speaker will have 15 minutes to present new data, provide relevant background information and historical context, and answer questions from the audience.

